# An Innovative Approach for Locating and Evaluating Subsurface Pathways for Nitrogen Loss

**DOI:** 10.1100/tsw.2001.286

**Published:** 2001-12-04

**Authors:** C.L. Walthall, T.J. Gish, C.S.T. Daughtry, W.P. Dulaney, K.-J.S. Kung, G. McCarty, D. Timlin, J. T. Angier, P. Buss, P. R. Houser

**Affiliations:** USDA-ARS Hydrology and Remote Sensing Lab, Beltsville, MD 20705, USA

**Keywords:** GIS, GPR, nitrogen, preferential flow, remote sensing, soil moisture sensors, subsurface flow, topography, yield monitoring

## Abstract

Fundamental watershed-scale processes governing chemical flux to neighboring ecosystems are so poorly understood that effective strategies for mitigating chemical contamination cannot be formulated. Characterization of evapotranspiration, surface runoff, plant uptake, subsurface preferential flow, behavior of the chemicals in neighboring ecosystems, and an understanding of how crop management practices influence these processes are needed. Adequate characterization of subsurface flow has been especially difficult because conventional sampling methods are ineffective for measuring preferential flow of water and solutes. A sampling strategy based on ground-penetrating radar (GPR) mapping of subsurface structures coupled with near real-time soil moisture data, surface topography, remotely sensed imagery, and a geographic information system (GIS) appears to offer a means of accurately identifying subsurface preferential flow pathways. Four small adjacent watersheds draining into a riparian wetland and first-order stream at the USDA-ARS Beltsville Agricultural Research Center, Beltsville, MD are being studied with this protocol. The spatial location of some preferential flow pathways for chemicals exiting these agricultural watersheds to the neighboring ecosystems have been identified. Confirmation of the pathways is via examination of patterns in yield monitor data and remote sensing imagery.

## INTRODUCTION

Excess agricultural chemical flux from crops can eventually contaminate surface and/or subsurface waters. Over 10% of the community wells nationwide have been contaminated with pesticides and over 50% of the drinking water wells contain nitrate[1]. Eliminating or reducing the effects of agricultural chemicals on water quality will safeguard human health, improve environmental quality, and reduce farm economic losses. This is a priority for areas such as the Chesapeake Bay watershed that rely on riparian zones as a buffer for agricultural groundwater nitrogen[2]. Unfortunately, the fundamental watershed-scale processes governing the dynamics of chemical transport through soil to neighboring ecosystems are so poorly understood that strategies for mitigating chemical contamination cannot be formulated. The major limitation to accurately quantifying and predicting chemical transport at the field and watershed scale is inadequate characterization of surface and subsurface water dynamics. Since significant movements of nitrogen are believed to occur with movements of water, a better understanding of nitrogen flux can be gained by characterizing watershed-scale water flux[3].

Both matrix flow and preferential flow processes govern water and chemical transport. Although matrix water flow processes are well understood, our knowledge of preferential flow is inadequate. Roth et al.[4] found that a uniformly-applied chemical pulse in a layered soil profile was split into a fast moving preferential flow front and a slow moving matrix flow front. The preferential flow front reached a depth of 2.3 m after only 3.1 cm of natural precipitation, while the matrix front moved 0.84 m after 85.3 cm of net infiltration. Kladivko et al.[5] found high concentrations of pesticides in water samples collected from tile drains buried 75 cm deep in a silty loam soil after only 1 cm of net infiltration. Flury et al.[6] used a soluble dye to stain flow pathways in 14 different soils and found movement beyond 1 m with only 4 cm of dyed water applied. It was concluded from this that preferential flow processes were dominant over matrix flow processes. Recently an innovative approach for measuring a chemical flux was developed and evaluated under field conditions at several locations in the U.S.[7,8]. This approach demonstrated that under moist, unsaturated soil conditions, as little as 1.3 cm of irrigation water could transport as much as 20% of a surface-applied mobile tracer to a depth of 1 m after 6 h, with a low irrigation intensity of 3 mm/h. Measures of classical saturated hydrologic parameters from the same fields indicated that it should have taken a minimum of at least 2 days for the soluble chemical to reach 1 m. These studies show that (1) under field conditions, preferential flow is a critical component of the total chemical flux; (2) real-time monitoring of preferential flow pathways is necessary if watershed-scale fluxes are to be accurately located, monitored, and ultimately controlled; (3) the timing of sample collection is critical in monitoring chemical leaching; and (4) random temporal collection of samples at only a few discrete times is inadequate for characterizing water and chemical transport.

Identifying and characterizing preferential flow is challenging. Kung et al.[7,8] developed an alternative method for determining chemical flux caused by preferential flow in tile-drained fields by applying and tracking chemicals in a narrow test strip of soil. Unfortunately, the technique cannot be widely adopted because most U.S. agriculture is not tile drained. Additionally, the formation of subsurface flow pathways, such as those below 1 m along the East Coast Piedmont Plateau, are often dictated by pedological and geologic events that have formed clay or sand lenses of various sizes, orientations, and thicknesses. These lenses and/or textural discontinuities can restrict and focus water into discrete subsurface flow pathways — much like valleys focusing rainwater into surface streams. Water percolating through the soil is eventually intercepted and then flows along these lenses and discontinuities (i.e., "funnel flow"). Although shallow subsurface flow pathways are temporally and spatially dynamic due to interactions with climate, management practices, and biophysical factors, the locations of the deeper subsurface pathways resulting from clay lenses or other restricting layers are probably static. Thus, it should be possible to detect and monitor watershed scale fluxes of water and solutes leaving agricultural landscapes and entering adjacent ecosystems if the location, size, shape, and orientation of the subsurface restricting layers can be characterized.

GPR can provide image profiles of soil subsurface stratigraphy and, hence, subsurface flow pathways. GPR delineates changes in the spatial continuity of subsurface dielectric properties and is useful for identifying soils with different water holding capacities[9,10,11]. Rea and Knight[12] found that GPR profile images showed the spatial continuity of coarse- and fine-grained beds of sedimentary deposits. GPR data have also been used to identify gross subsurface stratigraphies[13,14]. Donohue and Kung[15] exploited the capacity of GPR data to reveal textural discontinuities for the installation of suction lysimeters along potential preferential flow pathways. Solution samples collected from these lysimeters had solution volumes and chemical concentrations 400% larger than samples obtained where matrix flow was expected.

With knowledge of the subsurface flow pathways via GPR, a better determination of where and when to take hydrologic measurements in order to account for matrix and preferential flow processes can be made. The primary objective of this research is to determine if a protocol primarily based on GPR techniques can be developed for finding and monitoring subsurface flow pathways. A confirmation of the flow pathways will be determined by comparing the location of the flow pathways with surrogate indicators such as color infrared (CIR) remote sensing imagery and crop yield data, and directly by real-time soil moisture observations. The insights and data gained from this project can also be used to develop more accurate water quality simulation models. With the subsurface flow pathways identified, recommendations or management practices for reducing the subsurface chemical fluxes can eventually be explored.

## EXPERIMENTAL METHODS

The research site is a 20 ha field located in Beltsville, MD containing four small watersheds ranging from 3.4 to 4.1 ha each (Fig. 1). Each watershed is delimited by earthen berms planted to grass. The site was formed from sandy fluvial deposits and has a varying slope ranging from 1 to 4%. The soils are variable, with the majority being typic hapludults, coarse-loamy, siliceous, mesic. The soil series with the proportional extent of the respective mapping units is as follows: Downer-Muirkirk-Matawan sandy loam, 49%; Bourne fine sandy loam, 23%; Matawan-Hammonton loamy sand, 23%; and Downer-Ingleside loamy sand, 5%. Over 1700+ soil cores have been removed to characterize texture, pH, P content, organic matter content, and soil nitrogen concentration. We also measured (1) landscape geophysical properties using GPR, EM-31, EM-38, and multidepth and multifrequency EM; (2) surface hydrology using intensive real-time soil moisture monitoring, meteorological data, and surface runoff

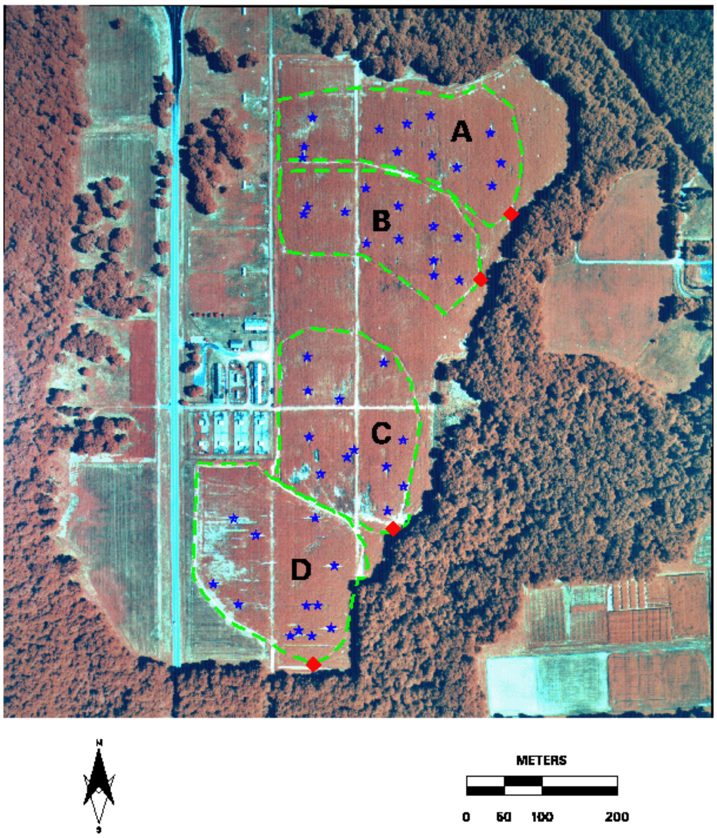

**FIGURE 1.** Color infrared aerial photography of the research site showing watershed designations and boundaries (dotted green lines), soil moisture stations (blue stars), surface runoff flumes (red diamonds), and forested riparian wetland into which each watershed drains. The riparian wetland contains a first-order stream with five water-flow monitoring stations.

fluxes of water, nitrate and pesticides; (3) pesticide vapor fluxes; and (4) various crop physiology and yield parameters. The site is equipped with two meteorological stations that continuously measure solar radiation, rainfall, vapor pressure, air temperature, and soil heat flux. The adjacent riparian wetland is equipped with 120 observation wells and 5 in-stream channel weirs for monitoring water table heights and stream flows, respectively. The watersheds have the highest elevation within this geographical area and so have no subsurface waters entering them from above the clay lenses for the top 5 m.

Only three of the four watersheds (A, B, and C in Fig. 1) were used for this analysis. A subsurface interface radar (SIR) system was used to acquire the georeferenced GPR data following calibration, on both coarse- and fine-resolution sample grids. The coarse-resolution sample grid of north/south transects spaced 25 m apart was used for gross characterization of the subsurface stratigraphy across the entire experimental field site. The fine resolution sample grid of north/south transects spaced 2 m apart, was used to locate soil moisture monitoring stations on 44 blocks of 25 × 25 m (0.06 ha) selected by stratified random design. Prior to interpretation, the GPR data were distance normalized to conform to known surface distances, and subsurface reflections were accentuated using a low pass filter. The digital trace in the profile image followed the first (i.e., shallowest) continuous feature that exhibited the strongest dielectric discontinuity. Strong dielectric reflections were considered to be a manifestation of soil textural discontinuities such as clay lenses restricting subsurface water flow. The fine grid GPR data provided nearly contiguous image profiles of the subsurface stratigraphy for precise installation of 256 real-time soil moisture sensors installed on 48 capacitance probes[16].

The spatial distribution of surface (0 to 30 cm) soil texture, organic matter content and phosphorous and potassium concentrations were determined by extracting soil cores on a 30- × 30-m soil sampling grid which was augmented by six additional transects (195 m long) sampled at a 15-m intervals. Six to eight soil cores were combined to produce a bulked sample at each of these 274 sample locations.

The GPR and soil core data sets were kriged. Contour and 3D surface maps of the depth to the first continuous restricting layer were brought into a GIS to overlay maps of other soil and crop parameters on the restricting layer maps. The depth to the first continuous restricting layer was subtracted from photogrammetrically derived surface elevations to produce subsurface restricting layer topography. Simple GIS-based hydrologic models were run on the data layers of the subsurface topography to determine potential convergent flow pathways (ArcInfo Flow Accumulation and Flow Direction). The potential convergent flow pathways and soil core data were compared to crop growth patterns on CIR imagery and to the spatial distribution of the corn grain yield. The soil moisture observations generate a localized view of soil water dynamics, while the corn grain yield and remote sensing data generate information on the areal extent of soil water availability, especially during drought years.

## RESULTS AND DISCUSSION

Fig. 2 shows the subsurface flow pathways for three of the watersheds. This is the first use of GPR to determine subsurface stratigraphy at this scale of observation (>4 ha). The subsurface restricting layers, typically a clay lens, on the study site appear to occur between 0.9 and 3.5 m below the soil surface.

Each soil moisture sensor is activated every 10 min, generating over 36,800 volumetric water contents daily over the entire research site. Because of space limitations, only one probe located near one of the GPR-identified subsurface flow pathways will be discussed. This particular probe contains six sensors at 0.1, 0.3, 0.5, 1.2, 1.5, and 1.8 m, and was installed where the GPR identified a lens at 1.54 m (Fig. 3).

The soil moisture data is divided into near surface (0.1 to 0.5 m) and subsurface (1.2 to 1.8 m) processes to better visualize water dynamics. The rapid rise of surface water contents during rain events and diurnal variations of water content at the surface (10 cm) evidence the sensitivity of these soil moisture sensors. These results include bare soil evaporation and perhaps soil temperature effects on the volumetric water content (DOY 133–144). During this same time period, dramatic changes occur in the water contents of the subsurface soil. Within a few hours the volumetric water contents rise from 0.08 to over 0.31 cm$^3$ water cm$^{-3}$ soil at 1.5 m before the water reaches 1.2-m depth. Such a large water plume could not originate from above. If the water plume had come from above, the water contents at 1.2 m would have increased before those at 1.5 m and would have eventually had similar volumetric water contents (note that 1.2- and 1.5-m depths have nearly identical coarse sandy soil with gravel textures). However, the water contents at 1.5 m not only rise before those at 1.2 m, but also are twice the magnitude of those observed at 1.2 m. The subsurface water is believed to be moving horizontally, riding atop the clay lens located at 1.54 m. This horizontal water plume kept volumetric water content of the coarse sand layer immediately above the clay lens over 0.30 cm$^3$ water cm$^{-3}$

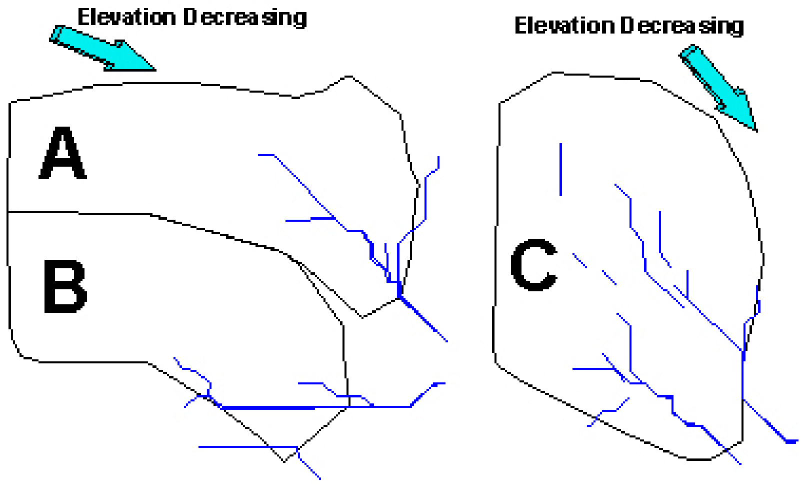

**FIGURE 2.** Location of GPR-identified subsurface flow pathways relative to watershed boundaries. Blue lines indicate regions of subsurface flow accumulation and direction.

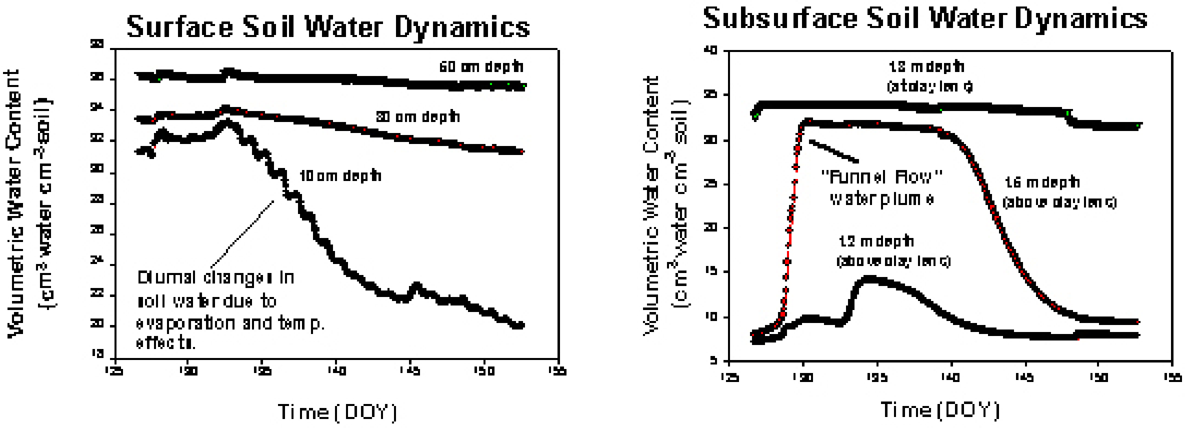

**FIGURE 3.** Volumetric water contents for a single soil moisture probe showing preferential funnel flow along a clay lens located at 1.54-m depth. The probe is located near a GPR subsurface flow pathway that was identified using a GIS.

soil for 12 days. The rise in water contents at the 1.2 m depth are probably due to the capillary rise of water originating from the horizontal water plume flowing along the clay lens, while the high water contents in the clay lens are due to the higher water holding capacity for clay. The significance of this process could easily be overlooked or misinterpreted if data collection took place only at weekly intervals instead of the nearly continuous monitoring.

Water and nutrients are believed to be congregated along subsurface convergent flow pathways throughout the field where soil moisture sensors are lacking. As a result, crop response to water and nutrients as increased yield and increased foliage density may be visible in yield monitor data and CIR imagery. Identification of these patterns can be useful for confirming the spatial expressions of subsurface flow pathways. This would be especially significant during drought years and if the subsurface flow paths are too distant from the active root zone.

The spatial distribution of corn grain yields are shown in Fig. 4 (**I** and **II**). These maps appear to provide beneficial insights, as 1998 and 1999 were drought years and the site lacks irrigation. Although water availability was more limiting in 1999 than in 1998, yield patterns from these 2 drought years show a marked degree of similarity, especially in the higher grain producing areas.

The data for the 2 years was averaged to determine if the regions of high yield appeared consistent (Fig. 4, **III**). The darker areas of the average yield maps represent areas that generate consistently high yields during drought years. Given normalization such as this, regions of the watershed having the greatest production consistently can be identified, isolated, and subsequently compared to other soil parameters using a GIS. For example, the organic matter distribution from the 1700+ soil cores across the 274 locations is shown in Fig. 4 (**IV**) along with regions having the highest yields for 2 consecutive moisture-limiting years. Note that areas with the highest yield (hatched polygons) occur in both low and high organic matter regions. Thus, highest yielding and highest organic matter content are not correlated. Similar poor correlations with yield were evident with sand, silt, or clay content distributions as well as phosphorous, potassium, and pH distributions.

The highest yielding areas of each watershed can also be compared to georectified CIR imagery and the GPR-identified flow pathways using a GIS. Unlike the spatial distribution of many soil physical and chemicals properties, there appear to be relationships between the crop growth and crop yield and the GPR-identified flow pathways (Fig. 5).

Areas with dark red tones on the CIR image indicate vigorous vegetative growth, while white and blue tones indicate nearly bare soil. The low vegetation amounts on the berms and the abandoned roads are clearly visible. The dark red regions within the watersheds appear to correspond to those regions of the watersheds with consistently high yields during the 1998 and 1999 “water limiting” seasons (black dotted polygons). Note that some areas of the watershed may have vigorous vegetative growth, yet lack high grain yields.

The subsurface flow pathways (blue lines) appear to be associated with the highest yielding areas of the watersheds (dotted black polygons). The GPR-identified subsurface pathways connect or pass between the highest yielding areas of each watershed, suggesting that those discrete subsurface flow pathways are real and perhaps serve as a subsurface irrigation system during drought conditions.

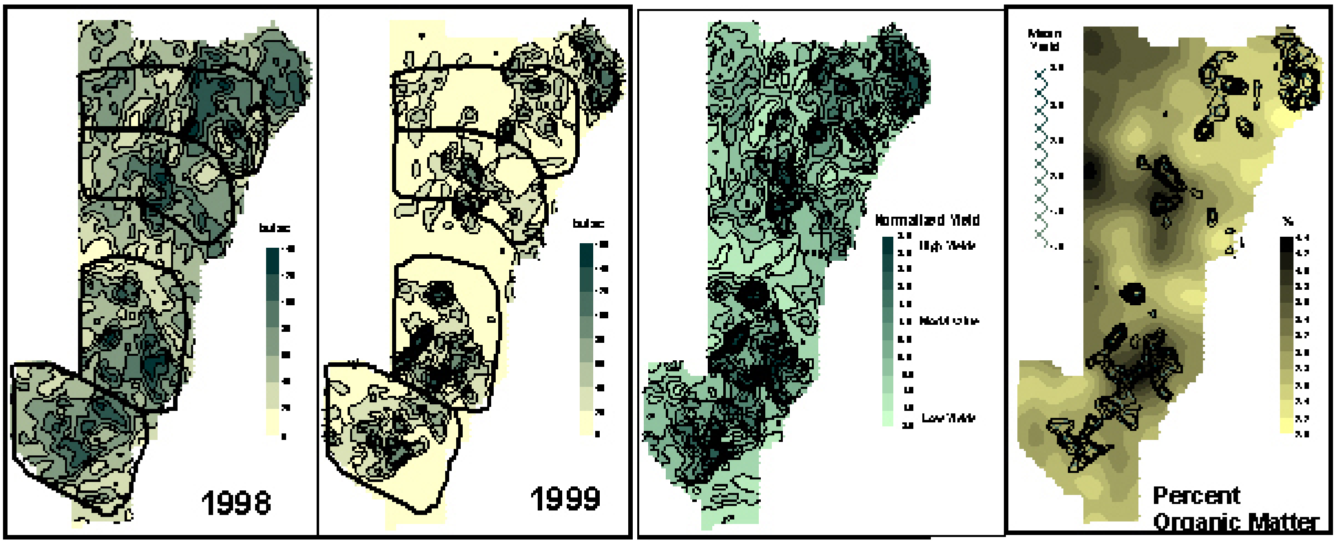

**FIGURE 4.** Spatial distribution of corn grain yields in 1998 and 1999 (**I** and **II**), averaged normalized yield (**III**), and comparison of high yielding areas with organic matter spatial distribution (**IV**).

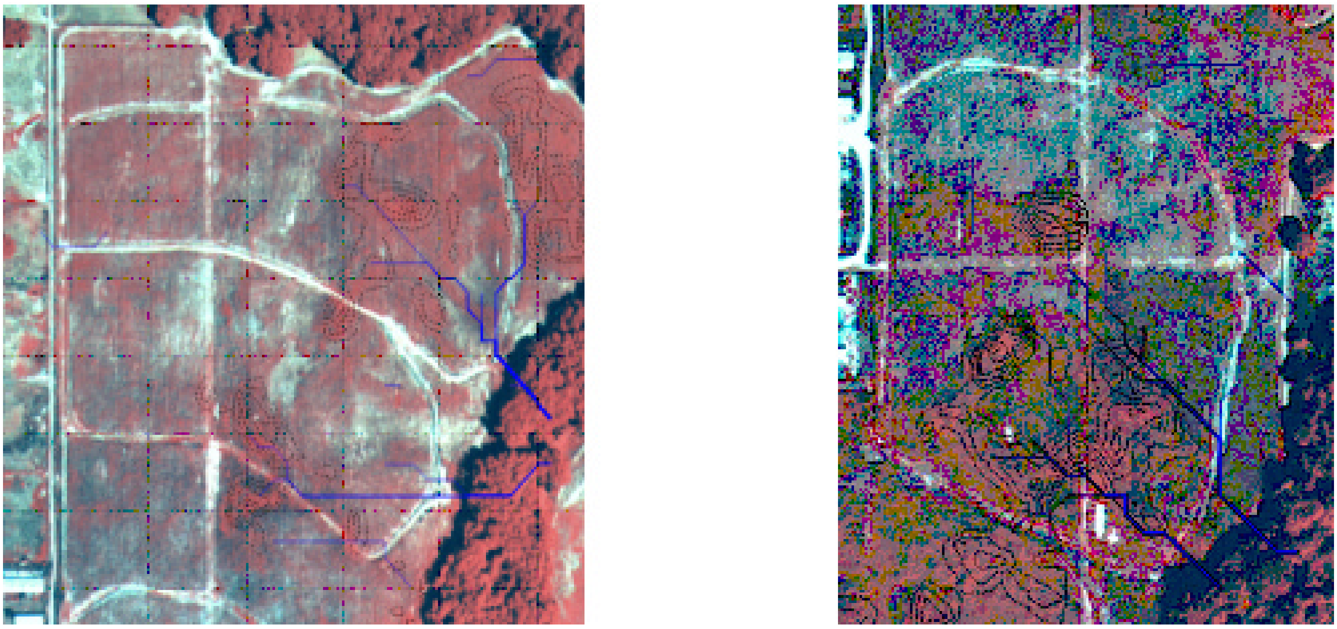

**FIGURE 5**. Visual correlation of highest yielding areas (black dotted polygons) with GPR-identified subsurface flow pathways (blue lines) and color infrared imagery. Watersheds A and B (**I**) and watershed C (**II**).

## CONCLUSIONS

Preferential flow is a critical element of water and chemical flux. A protocol for identifying preferential subsurface flow pathways can be combined with protocols for matrix flow, surface runoff, and vapor loss fluxes for accurate estimates of these fluxes. Use of GPR with a DEM, near real-time soil moisture monitoring, and widely available GIS routines can be used to identify preferential flow pathways. Confirmation of the preferential pathways with yield maps, remote sensing imagery, and soil moisture probes in the analysis reported here suggests that perhaps the CIR imagery and yield maps may provide surrogate indicators of flow pathways that can be used to guide GPR and soil moisture probe sampling. This will allow more accurate monitoring of water and chemical transport at the small watershed scale with more efficient sampling, and will ultimately permit the development of agricultural practices and/or recommendations that can reduce total daily maximum loads while maintaining farm profitability.

Additional tests of the protocols reported here are needed. Further investigations will be conducted in concert with other long-term goals of this project, which include (1) determining atmospheric, surface and subsurface watershed-scale fluxes of water, plant nutrients, and pesticides from three crop production systems; (2) determining the behavior and environmental impact of chemical inputs on a wooded riparian wetland and first-order stream; (3) developing remote sensing products and analytical techniques for measuring and managing the spatial variability of crops and soils; and (4) determining long-term economic and environmental impacts from three crop production systems and an evaluation of tradeoffs using integrated economic and biophysical simulation models.

## DISCLAIMER

Manufacturer names are provided for information purposes only and do not imply endorsement to the exclusion of others.

| | | |
|---|---|---|
| Received: | July | 20, 2001 |
| Revised: | October | 1, 2001 |
| Accepted: | October | 9, 2001 |
| Published: | December | 4, 2001 |

## BIOSKETCHES

**Charles L. Walthall** is a Research Physical Scientiest with the USDA Agricultural Research Service Hydrology and Remote Sensing Laboratory. He has a Ph.D. from the University of Nebraska in Agricultural Meteorology and Climatology/Agronomy with a specialization in remote sensing, an M.S. from Texas A&M in Forest Science with a specialization in remote sensing, and a B.S. in geography from the University of Maryland. His research is focused on furthering remote sensing science and remote sensing applications to agricultural problems. He was previously employed by the University of Maryland and NASA Goddard Space Flight Center.

**Timothy J. Gish** is a Soil Physicist with the USDA-ARS Hydrology and Remote Sensing Laboratory, Beltsville, Maryland. He received a B.S. from Brigham Young University, Provo, Utah and a Ph.D. from the University of California, Riverside. For the past two decades he has been a lead scientist in understanding preferential fluid dynamics and how this flow process influences field scale chemical behavior.

**Craig S.T. Daughtry** is a Research Agronomist with the Hydrology and Remote Sensing Laboratory of the USDA Agricultural Research Service in Beltsville, Maryland. He has B.S. and M.S. degrees from the University of Georgia and a Ph.D. from Purdue University. His research interests include developing methods to assess the condition and spatial variability of crops and soils for precision farming applications using remote sensing techniques.

**Wayne P. Dulaney** is a Remote Sensing Specialist with the USDA Agricultural Research Service. Upon completing degrees in Botany (B.S.) and Geography (M.A.) from the University of Maryland, College Park, he participated in investigations on the use of near-surface, remotely sensed observations for the estimation of surface energy balance components. More recent efforts have focused on the use of geospatial technologies such as remote sensing, GPS, GIS, and spatial statistics to assess crop growth variability for precision farming applications as well as the use of spaceborne, synthetic aperture radar (SAR) data to address agricultural issues at larger spatial scales.

**King Jau Samuel Kung** is a Soil Physicist in the Department of Soil Science, University of Wisconsin–Madison. He has a B.S. degree from Chung-Hsing University, Taiwan and M.S. and Ph.D. degrees from Cornell University, Ithaca, New York. Although Dr. Kung has many research interests, his primary focus is on understanding and characterizing water and chemical transport through soil. For the first time, he recently developed a procedure for accurately measuring a total chemical flux (including preferential flow) on undisturbed tile-drained fields.

**Dennis Timlin** is a soil scientist with the U.S. Department of Agriculture's Agricultural Research Service. He received his Masters and Ph.D. degrees from Cornell University. Dr. Timlin's study area is in the field of Soil Physics and his research interests are in the areas of water and solute transport in soil, developing mechanistic models of plant development and soil processes, bio-physical relationships between plants and soils, estimating soil hydraulic properties and understanding their relationships with landscape properties, and applying simulation models at landscape scales and with remotely sensed data.
